# The risk of withdrawal from hypertension treatment in coastal areas after the Great East Japan Earthquake: the TMM CommCohort Study

**DOI:** 10.1038/s41440-023-01454-0

**Published:** 2023-10-13

**Authors:** Rieko Hatanaka, Naoki Nakaya, Mana Kogure, Kumi Nakaya, Ippei Chiba, Ikumi Kanno, Hideaki Hashimoto, Tomohiro Nakamura, Kotaro Nochioka, Taku Obara, Yohei Hamanaka, Junichi Sugawara, Tomoko Kobayashi, Akira Uruno, Eiichi N. Kodama, Nobuo Fuse, Shinichi Kuriyama, Atsushi Hozawa

**Affiliations:** 1 Tohoku Medical Megabank Organization, Tohoku University, Aoba-ku, Sendai, Miyagi Japan; 2 Graduate School of Medicine, Tohoku University, Aoba-ku, Sendai, Miyagi Japan; 3 Tohoku University Hospital, Tohoku University, Aoba-ku, Sendai, Miyagi Japan; 4 International Research Institute of Disaster Science, Tohoku University, Aoba-ku, Sendai, Miyagi Japan

**Keywords:** Degree of damage by earthquake, Withdrawal, Treatment for hypertension, Great East Japan Earthquake, Cardiovascular disease prevention

## Abstract

This study aimed to examine whether risk of withdrawal from HTTx was higher in coastal areas that were severely damaged by tsunami than in inland areas. We conducted a cross-sectional study of 9218 participants aged ≥20 years in Miyagi, Japan. The odds ratios (ORs) and confidence interval (CI) for withdrawal from HTTx in coastal and inland groups were compared using multivariate logistic regression analysis, adjusting for potential confounders. In total, 194 of 5860 and 146 of 3358 participants in the inland and coastal groups, respectively, withdrew from HTTx treatment. OR (95%CI) of withdrawal from HTTx in the coastal group was 1.46 (1.14–1.86) compared to the inland group. According to housing damage, ORs (95% CI) in the no damage, partially destroyed, and more than half destroyed coastal groups compared with the no damage inland group were 1.62 (1.04–2.50), 1.69 (1.17–2.45), and 1.08 (0.71–1.65), respectively. In conclusion, the risk of HTTx withdrawal for participants whose homes in coastal areas were relatively less damaged was significantly higher compared with those in inland areas, while the risk of HTTx withdrawal for participants whose homes were more than half destroyed was not. Post-disaster administrative support for disaster victims is considered vital for continuation of their treatment.

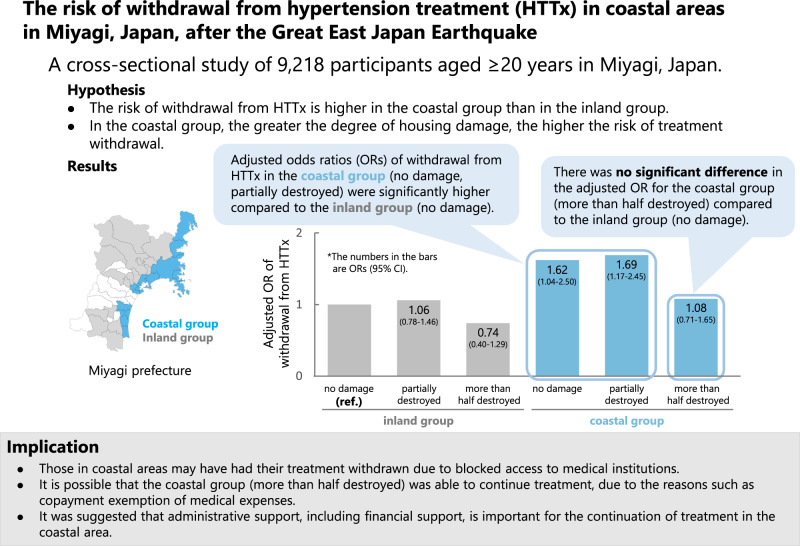

## Introduction

The 9.0-magnitude earthquake and tsunami that occurred on March 11, 2011 triggered the Great East Japan Earthquake (GEJE), causing extensive damage along Japan’s northeastern coast. As of June 10, 2021, 15,900 people have died [1], with drowning causing >90% of the deaths [2]. In addition to mental illness, psychological distress [3, 4], and pain [5, 6]; cardiovascular disease (CVD) risk factors, including increased incidence of hypertension [7], diabetes [8], and metabolic syndrome [9]; increased body weight, increased waist circumference, and decreased HDL-C [10]; as well as heart disease and cerebrovascular disease [11–13] have been reported in GEJE survivors. Therefore, concerns about the increase of CVD risk in the disaster area exist.

Many studies have reported an increased risk of CVD and death as blood pressure increases [14–17]. Poor adherence to medications including antihypertensives increases the risk of CVD and death [18, 19]. Therefore, taking appropriate medication and controlling blood pressure is important to prevent CVD onset.

A study involving residents in GEJE-affected coastal areas reported the association of psychological distress with an increased risk of withdrawal from hypertension treatment (HTTx) [20]. A difference in patient behavior depending on the degree of damage (e.g., human damage and house damage) caused by natural disasters is possible; however, to our knowledge, there are no reports comparing the risk of HTTx withdrawal in inland areas with that in coastal areas severely damaged by the tsunami. We hypothesized that coastal residents were at a greater risk of HTTx withdrawal than inland residents. To the best of our knowledge, this study pioneers on this topic. Analysis was performed on ~10,000 hypertensive participants requiring treatment.

## Methods

### Study design, setting, and participants

This is a cross-sectional study based on the Tohoku Medical Megabank Community-Based Cohort Study (TMM CommCohort Study) [21]. The TMM CommCohort study is a population-based cohort study designed to assess the long-term health effects of the GEJE and evaluate the gene–environment interactions in the onset of major diseases such as cardiovascular disease and cancer.

The baseline survey of the TMM CommCohort Study was conducted from May 2013 to March 2016 among persons aged ≥20 years residing in Miyagi and Iwate prefectures. It included three types of surveys: Type 1 (*n* = 40,433), Type 1 additional (*n* = 664), and Type 2 surveys (*n* = 13,855). The Type 1 survey was conducted at the sites of specific health checkups in the municipalities. In the Type 1 survey, the following basic information was collected: blood sample, urine sample, questionnaires, and municipal specific health checkup data. The Type 1 additional survey was conducted on a different date from the municipal specific health checkup with ToMMo and the municipalities coordinating the date and locations.

The Type 2 survey used mass media to advertise for enrollment. In addition to the information collected in the Type 1 survey and the Type 1 additional survey, this survey also collected detailed measurement regarding body composition, respiratory function, and carotid ultrasound imaging. This survey was conducted at assessment centers—the Community Support Center in Miyagi and Satellite in Iwate.

This study included 40,433 participants living in Miyagi who completed the Type 1 survey. We excluded those who withdrew consent as of October 5, 2021 (*n* = 1428), and 39,005 participants were involved in the study.

Among those participants, 36,652 responded to the questionnaire. In total, 9746 of the respondents answered “I have been diagnosed as having hypertension, and I am currently under treatment” or “I have been diagnosed as having hypertension, and I am withdrawal this treatment” in response to a question about the treatment status of hypertension.

Those with missing data on the degree of their housing damage, place of residence (inland or coastal area), systolic blood pressure (SBP), diastolic blood pressure (DBP), and body mass index (BMI) were excluded (*n* = 528). Finally, 9218 participants were included in the study.

This study was approved by the Institutional Review Board of the Tohoku Medical Megabank Organization (approval number: 2022-4-126). All participants provided written informed consent.

### Outcome measurement: the treatment status of hypertension

The treatment status of hypertension at the baseline survey was obtained using a self-administered questionnaire. The questionnaires were handed to the participants at the site of the specific municipal health checkup, and the completed questionnaires were returned to our research center by mail.

The participant answered by selecting one of the following 5 items: (1) “I have been diagnosed as having hypertension, and I am currently under treatment,” (2) “I have been diagnosed as having hypertension and I am withdrawal this treatment,” (3) “I have been diagnosed as having hypertension, and I’m only careful about my lifestyle,” (4) “I have been diagnosed with hypertension, but I am currently following up without this treatment according to the doctor’s instructions,” or (5) “I have never been diagnosed with hypertension.”

In this study, participants who answered (3)–(5) were excluded, thereby leaving only (1) and (2). Participants who answered (1) and (2) were defined as “under HTTx” and “withdrawal from HTTx,” respectively.

### Exposure measurement

#### Residential area (inland areas or coastal areas)

The participants were classified into two residential areas, inland and coastal, according to their address information at the time of the baseline survey (extracted on October 14, 2020). Details are shown in Supplementary Fig. 1.

#### The degree of housing damage

The degree of housing damage was obtained by responding to the self-administered questionnaire. The participants selected one of the following items regarding their degree of housing damage at the time of the GEJE. The items were: “totally destroyed (totally destroyed outflow),” “large-scale destroyed,” “half-destroyed,” “partially-destroyed,” “no damage,” or “not living in the disaster area,” We have divided these items into three categories: “totally destroyed,” “large-scale half-destroyed,” and “half-destroyed” together as “More than half destroyed,” and “partially destroyed” as “Partially destroyed,” “No damage,” and “No residence in the disaster area” were combined and “No damage.”

Even within the inland and coastal areas, there are differences in the degree of housing damage. Hence, the participants who lived in inland and coastal areas were further divided into three groups according to the damage to their homes.

### Variables

Age and sex were obtained from the information provided in the consent form. The following information was obtained from a self-administered questionnaire: smoking status, drinking status, psychological distress assessed using the K6 score, social network status assessed using the Lubben Social Network Scale-6 (LSNS-6), educational background, having a spouse, having children, living with family, ever been diagnosed with diabetes, ever been diagnosed with dyslipidemia, and employment status. The year of the examination at the specific health checkup site was used as the fiscal year of survey participation (FY2013, FY2014, and FY2015). Missing values were defined as “unknown.”

Smoking status was classified as “current smoker” if the number of cigarettes smoked from birth to date was 100 or more and the person was currently smoking, “ex-smoker” if the number of cigarettes smoked from birth to date was 100 or more but the person did not currently smoke, and “never smoker” if the number of cigarettes smoked from birth to date was less than 100.

If the participants answered “yes” to the question “Do you drink alcohol?”, they were categorized as “current drinker,” if they answered “quit,” they were categorized as “ex-drinker,” and if they answered “hardly (not at all) drinkers” or “constitutionally unable to drink,” they were categorized as “never drinker.”

A K6 score of ≥5 was defined as having psychological distress [22–24]. If the LSNS-6 score was <12 points, the participants were defined as socially isolated [25, 26]. The estimated daily salt intake was calculated using the Tanaka formula [27], as follows:

$${{{{{{\rm{Estimated}}}}}}\,24{{{{{\rm{HUNaV}}}}}}[{{{{{\rm{mEq}}}}}}/{{{{{\rm{day}}}}}}]=21.98\times {{{{{\rm{XNa}}}}}}}^{0.392}$$

$${{{{{\rm{PRCr}}}}}}[{{{{{\rm{mg}}}}}}/{{{{{\rm{day}}}}}}]= 	-2.04\times {{{{{\rm{age}}}}}}+14.89\times {{{{{\rm{weight}}}}}}\,[{{{{{\rm{kg}}}}}}]\\ 	+16.14\times {{{{{\rm{height}}}}}}\,[{{{{{\rm{cm}}}}}}]-2244.45$$

where XNa [mEq/day] = (SUNa [mEq/l]/SUCr [mg/dl]/10) × PRCr [mg/day]; 24HUNaV = 24-h urinary sodium excretion; PRCr = predicted value of 24-h urinary creatinine excretion; SUNa = Na concentration in the spot urine; SUCr = creatinine concentration in the spot urine.

The estimated daily salt intake was classified into two categories—above the median (>9.77) and below the median (≤9.77).

BMI was calculated using data from height and weight measured at the site of the specific municipal health checkup. SBP and DBP were measured with the participant in a sitting position using an upper arm automatic sphygmomanometer at the site of the specific municipal health checkup. Although blood pressure was measured once or twice per person, the first value was used for the analysis.

### Statistical analyses

Logistic regression analysis was used to calculate the odds ratios (ORs) and 95% confidence interval (CI) for withdrawal from HTTx compared with that of the entire inland group, or the inland (no damage) group. Covariates used in the multivariate logistic regression analysis included age, sex, the fiscal year of survey participation, smoking status (current smoker, ex-smoker, never smoker, unknown), drinking status (current drinker, ex-drinker, never drinker, unknown), having psychological distress (yes, no, unknown), social network scale (socially isolated, socially connected, unknown), educational background (elementary/junior high/high school, vocational school/junior college/technical college, university or higher, other, unknown), having a spouse (yes, no, unknown), having children (yes, no, unknown), living with family (yes, no, unknown), ever been diagnosed with diabetes, (yes, no, unknown), ever been diagnosed with dyslipidemia, (yes, no, unknown), employment status (employed, unemployed, unknown), and estimated daily salt intake (above the median, below the median, unknown).

Next, to examine whether the proportion of hypertensives differed among participants “under HTTx” and “withdrawal from HTTx,” we classified each subject into six categories based on the “Classification of blood pressure levels in adults” of the Japanese Society of Hypertension Guideline for the Management of Hypertension [28] and compared them using chi-square test. Furthermore, to compare whether the blood pressure levels differed among the participants “under HTTx” and “withdrawal from HTTx,” analysis of covariance was used to compare the SBP and DBP of the participants “under HTTx” and “withdrawal from HTTx,” respectively. Covariates used were age, sex, BMI, smoking status (current smoker, ex-smoker, never smoker, unknown), drinking status (current drinker, ex-drinker, never drinker, unknown), and estimated daily salt intake (above the median, below the median, unknown).

All statistical analyses were conducted using SAS version 9.4 (SAS Inc, Cary, NC). Two-tailed *p* values < 0.05 were considered statistically significant.

## Results

Table 1 shows the basic characteristics of the participants according to the place of residence (inland and coastal areas) and the degree of housing damage in the inland and coastal group. The percentage of participants who withdrew from HTTx was significantly higher in coastal areas compared to inland areas. Furthermore, the percentage of participants who withdrew from HTTx was higher in the no damage and partially damaged categories in the coastal group than in the inland group.

**Table 1:** Basic characteristics of the participants

	All		Inland area (all)		Inland area						Coastal area (all)		Coastal area					
					No damage		Partially destroyed		More than half destroyed				No damage		Partially destroyed		More than half destroyed	
	(*n* = 9218)		(*n* = 5860)		(*n* = 1924)		(*n* = 3378)		(*n* = 558)		(*n* = 3358)		(*n* = 707)		(*n* = 1394)		(*n* = 1257)	
Age, years	65.5	(6.3)	65.3	(6.3)	65.6	(6.4)	65.3	(6.1)	64.7	(6.4)	65.7	(6.4)	65.8	(6.7)	65.9	(6.1)	65.4	(6.7)
Sex																		
Male	4468	(48.5)	2874	(49.0)	896	(46.6)	1700	(50.3)	278	(49.8)	1594	(47.5)	328	(46.4)	675	(48.4)	591	(47.0)
Female	4750	(51.5)	2986	(51.0)	1028	(53.4)	1678	(49.7)	280	(50.2)	1764	(52.5)	379	(53.6)	719	(51.6)	666	(53.0)
BMI, kg/m^2^	24.8	(3.5)	24.7	(3.5)	24.8	(3.6)	24.7	(3.5)	24.8	(3.4)	24.8	(3.4)	24.6	(3.4)	24.7	(3.4)	25.1	(3.5)
SBP, mmHg	132.4	(15.8)	132.3	(15.7)	132.7	(15.9)	132.2	(15.6)	131	(15.9)	132.7	(16.0)	133.1	(15.8)	131.7	(16.5)	133.5	(15.5)
DBP, mmHg	78.7	(9.9)	78.8	(10.0)	78.6	(10.1)	79.0	(9.9)	78.6	(10.1)	78.5	(9.8)	78.9	(9.7)	78.0	(9.8)	78.9	(9.8)
Smoking status																		
Current smoker	1057	(11.5)	668	(11.4)	228	(11.9)	373	(11.0)	67	(12.0)	389	(11.6)	80	(11.3)	149	(10.7)	160	(12.7)
Ex-smoker	2779	(30.2)	1755	(30.0)	545	(28.3)	1034	(30.6)	176	(31.5)	1024	(30.5)	213	(30.1)	433	(31.1)	378	(30.1)
Never smoker	5142	(55.8)	3293	(56.2)	1113	(57.9)	1884	(55.8)	296	(53.1)	1849	(55.1)	396	(56.0)	764	(54.8)	689	(54.8)
Unknown	240	(2.6)	144	(2.5)	38	(2)	87	(2.6)	19	(3.4)	96	(2.9)	18	(2.6)	48	(3.4)	30	(2.4)
Drinking status																		
Current drinker	5129	(55.6)	3303	(56.4)	1037	(53.9)	1957	(57.9)	309	(55.4)	1826	(54.4)	395	(55.9)	790	(56.7)	641	(51.0)
Ex-drinker	287	(3.1)	186	(3.2)	63	(3.3)	103	(3.1)	20	(3.6)	101	(3.0)	19	(2.7)	37	(2.7)	45	(3.6)
Never drinker	3724	(40.4)	2316	(39.5)	803	(41.7)	1289	(38.2)	224	(40.1)	1408	(41.9)	291	(41.2)	557	(40.0)	560	(44.6)
Unknown	78	(0.9)	55	(0.9)	21	(1.1)	29	(0.9)	5	(0.9)	23	(0.7)	2	(0.3)	10	(0.7)	11	(0.9)
Having psychological distress																		
Yes	3378	(36.7)	2062	(35.2)	618	(32.1)	1225	(36.3)	219	(39.3)	1316	(39.2)	235	(33.2)	528	(37.9)	553	(44.0)
No	5666	(61.5)	3691	(63.0)	1276	(66.3)	2090	(61.9)	325	(58.2)	1975	(58.8)	463	(65.5)	841	(60.3)	671	(53.4)
Unknown	174	(1.9)	107	(1.8)	30	(1.6)	63	(1.9)	14	(2.5)	67	(2.0)	9	(1.3)	25	(1.8)	33	(2.6)
Social network status																		
Socially isolated	2090	(22.7)	1307	(22.3)	475	(24.7)	712	(21.1)	120	(21.5)	783	(23.3)	189	(26.7)	317	(22.7)	277	(22.0)
Socially connected	6709	(72.8)	4297	(73.3)	1365	(71.0)	2525	(74.8)	407	(72.9)	2412	(71.8)	489	(69.2)	1011	(72.5)	912	(72.6)
Unknown	419	(4.6)	256	(4.4)	84	(4.4)	141	(4.2)	31	(5.6)	163	(4.9)	29	(4.1)	66	(4.7)	68	(5.4)
Educational background																		
Elementary/junior high/high school	6686	(72.6)	4226	(72.1)	1417	(73.7)	2439	(72.2)	370	(66.3)	2460	(73.3)	519	(73.4)	1004	(72.0)	937	(74.5)
Vocational school/junior college/technical college	1650	(17.9)	1088	(18.6)	332	(17.3)	628	(18.6)	128	(22.9)	562	(16.7)	108	(15.3)	237	(17.0)	217	(17.3)
University or higher	787	(8.5)	495	(8.5)	161	(8.4)	280	(8.3)	54	(9.7)	292	(8.7)	70	(9.9)	138	(9.9)	84	(6.7)
Other	65	(0.7)	38	(0.7)	12	(0.6)	21	(0.6)	5	(0.9)	27	(0.8)	6	(0.9)	9	(0.7)	12	(1.0)
Unknown	30	(0.3)	13	(0.2)	2	(0.1)	10	(0.3)	1	(0.2)	17	(0.5)	4	(0.6)	6	(0.4)	7	(0.6)
Having a spouse																		
Yes	7629	(82.8)	4884	(83.3)	1565	(81.3)	2850	(84.4)	469	(84.1)	2745	(81.8)	574	(81.2)	1178	(84.5)	993	(79.0)
No	1516	(16.5)	934	(15.9)	346	(18)	503	(14.9)	85	(15.2)	582	(17.3)	129	(18.3)	204	(14.6)	249	(19.8)
Unknown	73	(0.8)	42	(0.7)	13	(0.7)	25	(0.7)	4	(0.7)	31	(0.9)	4	(0.6)	12	(0.9)	15	(1.2)
Having children																		
Yes	8453	(91.7)	5394	(92.1)	1737	(90.3)	3127	(92.6)	530	(95.0)	3059	(91.1)	650	(91.9)	1273	(91.3)	1136	(90.4)
No	275	(3.0)	177	(3.0)	68	(3.5)	99	(2.9)	10	(1.8)	98	(2.9)	19	(2.7)	49	(3.5)	30	(2.4)
Unknown	490	(5.3)	289	(4.9)	119	(6.2)	152	(4.5)	18	(3.2)	201	(6.0)	38	(5.4)	72	(5.2)	91	(7.2)
Living with family																		
Yes	8388	(91.0)	5370	(91.6)	1727	(89.8)	3125	(92.5)	518	(92.8)	3018	(89.9)	642	(90.8)	1270	(91.1)	1106	(88.0)
No	678	(7.4)	400	(6.8)	159	(8.3)	206	(6.1)	35	(6.3)	278	(8.3)	58	(8.2)	100	(7.2)	120	(9.6)
Unknown	152	(1.7)	90	(1.5)	38	(2.0)	47	(1.4)	5	(0.9)	62	(1.9)	7	(1.0)	24	(1.7)	31	(2.5)
Ever been diagnosed with diabetes																		
Yes	2009	(21.8)	1280	(21.8)	386	(20.1)	787	(23.3)	107	(19.2)	729	(21.7)	150	(21.2)	325	(23.3)	254	(20.2)
No	2892	(31.4)	1854	(31.6)	594	(30.9)	1074	(31.8)	186	(33.3)	1038	(30.9)	234	(33.1)	450	(32.3)	354	(28.2)
Unknown	4317	(46.8)	2726	(46.5)	944	(49.1)	1517	(44.9)	265	(47.5)	1591	(47.4)	323	(45.7)	619	(44.4)	649	(51.6)
Ever been diagnosed with dyslipidemia																		
Yes	2987	(32.4)	1801	(30.7)	558	(30.6)	1035	(30.6)	178	(31.9)	1186	(35.3)	246	(34.8)	530	(38.0)	410	(32.6)
No	2296	(24.9)	1525	(26.0)	481	(25.0)	907	(26.9)	137	(24.6)	771	(23.0)	177	(25.0)	320	(23.0)	274	(21.8)
Unknown	3935	(42.7)	2534	(43.2)	855	(44.4)	1436	(42.5)	243	(43.6)	1401	(41.7)	284	(40.2)	544	(39.0)	573	(45.6)
Employment status																		
Employed	3841	(41.7)	2640	(45.1)	799	(41.5)	1555	(46.0)	286	(51.3)	1201	(35.8)	227	(32.1)	491	(35.2)	483	(38.4)
Unemployed	5225	(56.7)	3141	(53.6)	1102	(57.3)	1776	(52.6)	263	(47.1)	2084	(62.1)	467	(66.1)	881	(63.2)	736	(58.6)
Unknown	152	(1.7)	79	(1.4)	23	(1.2)	47	(1.4)	9	(1.6)	73	(2.2)	13	(1.8)	22	(1.6)	38	(3.0)
Fiscal year of survey participation																		
FY2013	1728	(18.8)	583	(10.0)	209	(10.9)	309	(9.2)	65	(11.7)	1145	(34.1)	165	(23.3)	408	(29.3)	572	(45.5)
FY2014	4204	(45.6)	2400	(41.0)	737	(38.3)	1398	(41.4)	265	(47.5)	1804	(53.7)	444	(62.8)	759	(54.5)	601	(47.8)
FY2015	3286	(35.7)	2877	(49.1)	978	(50.8)	1671	(49.5)	228	(40.9)	409	(12.2)	98	(13.9)	227	(16.3)	84	(6.7)
Estimated daily salt intake, g/day																		
>9.77	4598	(49.9)	2936	(50.1)	941	(48.9)	1731	(51.2)	264	(47.3)	1662	(49.5)	357	(50.5)	689	(49.4)	616	(49.0)
≤9.77	4599	(49.9)	2916	(49.8)	981	(51.0)	1641	(48.6)	294	(52.7)	1683	(50.1)	348	(49.2)	699	(50.1)	636	(50.6)
Unknown	21	(0.23)	8	(0.1)	2	(0.1)	6	(0.2)	0	(0.0)	13	(0.4)	2	(0.3)	6	(0.4)	5	(0.4)
Treatment status of hypertension																		
Under HTTx	8878	(96.3)	5666	(96.7)	1862	(96.8)	3261	(96.5)	543	(97.3)	3212	(95.7)	671	(94.9)	1326	(95.1)	1215	(96.7)
Withdrawal from HTTx	340	(3.7)	194	(3.3)	62	(3.2)	117	(3.5)	15	(2.7)	146	(4.3)	36	(5.1)	68	(4.9)	42	(3.3)

The values in the table are presented as means (standard deviations) for continuous variables and as numbers (percentage) for categorical variables

*BMI* body mass index, *DBP* diastolic blood pressure, *HTTx* treatment for hypertension, *SBP* systolic blood pressure

Table 2 shows the risk of withdrawal from HTTx in the coastal group compared to the inland group. The ORs for withdrawal from HTTx were significantly higher in the coastal group compared to those in the inland group (OR 1.33, 95% CI 1.07–1.65). This association remained unchanged after adjustment for age, sex, the fiscal year of survey participation (Model 2: OR 1.44, 95% CI 1.13–1.83), and other confounding factors (Model 3: OR 1.46, 95% CI 1.14–1.86).

**Table 2:** Adjusted ORs (95% CIs) for withdrawal from HTTx in coastal areas

	Inland area (*n* = 5860)	Coastal area^a^ (*n* = 3358)
No. of participants under HTTx	5666	3212
No. of participants withdrawal from HTTx	194	146
Percentage of participants withdrawal from HTTx	3.3	4.3
Model 1: Crude OR (95% CI)	1.00 (Ref.)	1.33 (1.07–1.65)
* p* value		0.011
Model 2: Adjusted OR (95% CI)	1.00 (Ref.)	1.44 (1.13–1.83)
* p* value		0.003
Model 3: Adjusted OR (95% CI)	1.00 (Ref.)	1.46 (1.14–1.86)
* p* value		0.003

Model 1: unadjusted; Model 2: adjusted for age, sex, and fiscal year of survey participation; Model 3: adjusted for age, sex, fiscal year of survey participation, smoking status, drinking status, having psychological distress, social network status, educational background, having a spouse, having children, living with family, ever been diagnosed with diabetes, ever been diagnosed with dyslipidemia, employment status, and estimated daily salt intake

*CI* confidence interval, *OR* odds ratio, *HTTx* treatment for hypertension

^a^Odds ratios and 95% confidence intervals and *p* values for withdrawal from HTTx for the entire coastal area compared to those of the inland area are shown

Next, the inland and coastal groups were further divided into three groups according to the degree of housing damage. Adjusted ORs for withdrawal from HTTx were significantly higher in the no damage group (Model 3: OR 1.62, 95% CI 1.04–2.50) and partially destroyed group (Model 3: OR 1.69, 95% CI 1.17–2.45) living in coastal areas than in those living inland (Table 3). Adjusted ORs for withdrawal from HTTx in the coastal group (more than half destroyed) were similar to the no damage group in the inland (Model 3: OR 1.08, 95% CI 0.71–1.65).

**Table 3:** Adjusted ORs (95% CIs) for withdrawal from HTTx by residential area and degree of housing damage

	Inland area			Coastal area		
	No damage (*n* = 1924)	Partially destroyed^a^ (*n* = 3378)	More than half destroyed^a^ (*n* = 558)	No damage^a^ (*n* = 707)	Partially destroyed^a^ (*n* = 1394)	More than half destroyed^a^ (*n* = 1257)
No. of participants under HTTx	1862	3261	543	671	1326	1215
No. of participants who withdrew from HTTx	62	117	15	36	68	42
Percentage of participants who withdrew from HTTx	3.2	3.5	2.7	5.1	4.9	3.3
Model 1: Crude OR (95% CI)	1.00 (Ref.)	1.08 (0.79–1.48)	0.83 (0.45–1.43)	1.61 (1.05–2.44)	1.54 (1.08–2.19)	1.04 (0.69–1.54)
* p* value		0.640	0.522	0.026	0.016	0.854
Model 2: Adjusted OR (95% CI)	1.00 (Ref.)	1.06 (0.77–1.46)	0.77 (0.42–1.33)	1.64 (1.06–2.51)	1.64 (1.14–2.36)	1.09 (0.71–1.65)
* p* value		0.732	0.375	0.025	0.008	0.688
Model 3: Adjusted OR (95% CI)	1.00 (Ref.)	1.06 (0.78–1.46)	0.74 (0.40–1.29)	1.62 (1.04–2.50)	1.69 (1.17–2.45)	1.08 (0.71–1.65)
* p* value		0.719	0.312	0.03	0.005	0.708

Model 1: unadjusted; Model 2: adjusted for age, sex, and fiscal year of survey participation; Model 3: adjusted for age, sex, fiscal year of survey participation, smoking status, drinking status, having psychological distress, social network status, educational background, having a spouse, having children, living with family, ever been diagnosed with diabetes, ever been diagnosed with dyslipidemia, employment status, and estimated daily salt intake

*CI* confidence interval, *OR* odds ratio, *HTTx* treatment for hypertension

^a^Odds ratios, 95% confidence intervals, and *p* values for inland areas (partially destroyed), inland areas (more than half destroyed), coastal areas (no damage), coastal areas (partially destroyed), and coastal areas (more than half destroyed) compared to those of inland areas (no damage) are shown

**Fig. 1:**
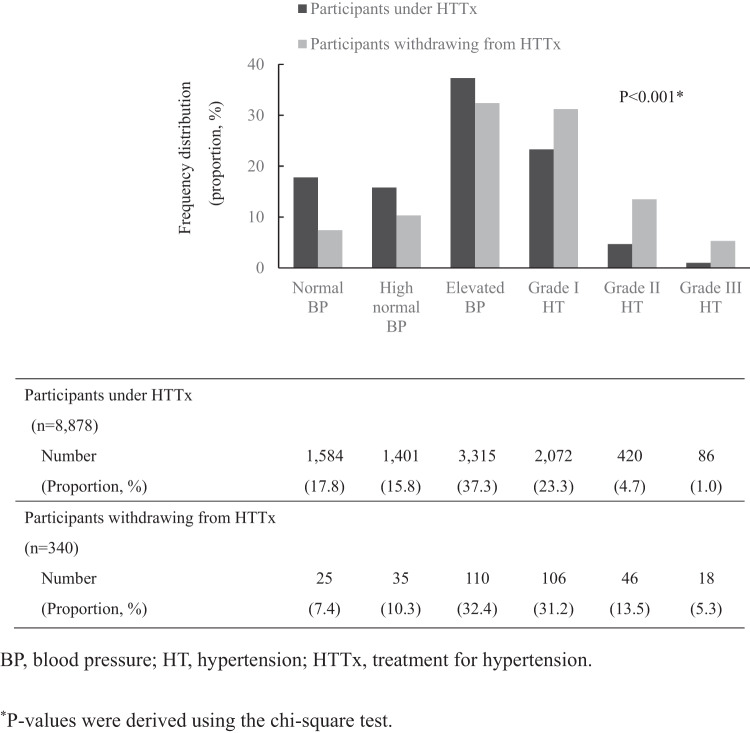
Frequency distribution of blood pressure levels in participants under HTTx (*n* = 8878) and in those withdrawing from HTTx (*n* = 340)

Figure 1 shows the frequency distribution of blood pressure levels in participants under HTTx and in those who withdrew from HTTx. For participants under HTTx and those who withdrew from HTTx, the distribution was skewed toward the group with lower and higher blood pressure values, respectively (*χ*^2^ test, *p* < 0.001).

Supplementary Table 1 shows a comparison of adjusted SBP and DBP between participants under HTTx and those who withdrew from HTTx. Participants who withdrew from HTTx had significantly higher SBP and DBP than those under HTTx (*p* < 0.001).

## Discussion

In this study, we found that the risk of withdrawal from HTTx was significantly higher in the coastal group than in the inland group. When the inland and coastal groups were further divided into three groups according to the degree of housing damage, the risk of withdrawal from HTTx in the coastal group (more than half destroyed) was similar to that of the no damage group in the inland. We also found that more participants who withdrew from HTTx had uncontrolled blood pressure levels (SBP ≥ 140 mmHg and/or DBP ≥ 90 mmHg) than those who were under HTTx.

A possible reason for the higher risk of withdrawal from HTTx in the coastal group compared to that of the inland group is that access to medical institutions was blocked in coastal areas. In Miyagi Prefecture, 128 of 147 hospitals and 624 of 1626 clinics (as of July 2011) [29] were totally or partially destroyed by the GEJE, and medical institutions, mainly in coastal areas, were severely damaged by the tsunami [30]. Furthermore, in the coastal areas of Miyagi Prefecture, many cars were washed away by the tsunami [30]; it was estimated that about 146,000 cars were damaged [31]. Many tsunami survivors lost their cars, which affects people’s daily lives long term, especially for the aged. Therefore, individuals residing in coastal areas may have withdrawn from treatment because they lost their means of transportation or because nearby medical institutions (e.g., their family doctor) were damaged and unable to provide medical services. Once treatment has been withdrawn, it remains withdrawn without resumption. Originally, a large proportion of the inland area of Miyagi Prefecture was medically underserved with inadequate transport; therefore, those living in inland areas are considered to be at high risk of withdrawal from HTTx. The fact that the risk of withdrawal from HTTx is higher in the coastal area than in the inland area indicates that the coastal area was severely damaged by the GEJE, and hence, coastal residents were unable to receive medical care.

An unexpected finding was a similar risk of withdrawal from HTTx between the coastal (more than half destroyed) and inland groups (no damage). One reason for this might be the effectiveness of government measures regarding medical expenses implemented after the earthquake. Those whose homes were damaged by more than half were exempted from a copayment of their medical bills, with public support from the government [32]. Although the period of copayment exemption differed depending on the type of health insurance, those insured by the National Health Insurance (NHI), which include most of the participants of this study, were exempted from paying over-the-counter copayments from the occurrence of the earthquake until at least September 2012, if they fulfilled certain requirements. After introducing this exemption measure, the number of health insurance claims (per month and per person) for medical outpatients for NHI reportedly increased [33]. The “more than half destroyed” coastal group may have been able to receive financial assistance, contributing to the reduction in withdrawal from treatment. Furthermore, in GEJE, ethical pharmaceuticals were transported to the three affected prefectures (Iwate, Miyagi, and Fukushima) and supplied to evacuation centers [34, 35]. It is possible that these supports kept participants who were “more than half destroyed” continue taking their medicines. Moreover, it is speculated that compared to people living in their own houses after the disaster, those whose houses were severely damaged and who had moved to evacuation shelters or temporary housing may have better access to medical institutions and pharmacies because of closer location, frequent availability of buses, and better roads, among others.

A higher percentage of participants who withdrew from HTTx had grade I hypertension or higher, and their BP levels were significantly higher than of those under HTTx (Fig. 1 and Supplementary Table 1). A recent meta-analysis reported that a 5 mmHg decrease in SBP was associated with an ~10% reduction in the risk of major cardiovascular events [36]. In this study, SBP differed by eight mmHg between those who withdrew from HTTx and those who were under HTTx. Therefore, the risk of CVD incidence in participants who withdrew from HTTx might potentially increase by ~16% compared to those under HTTx. Consequently, it is important to ensure that individuals requiring treatment continue to receive HTTx after a catastrophe.

### Limitations

This study has a few limitations. First, because the timing of withdrawal from HTTx (pre- or post-disaster) was unknown, some of the participants in this study might have withdrawn from treatment before the earthquake. However, it is unlikely that there was such a large difference between inland and coastal areas in this regard. Therefore, the presence of those who withdrew from treatment before the earthquake is unlikely to affect the association between the place of residence (inland or coastal) and the risk of withdrawal from HTTx. Second, the time relationship between the withdrawal of HTTx and BP measurements was not clear. Although BP was measured prior to completing the questionnaire, it is unclear whether the withdrawal from HTTx occurred before or after the BP measurements. However, the difference between the date of filling out the questionnaire and the date of BP measurement was 83.0% and 90.7% within 14 and 30 days, respectively (*n* = 8814). It is unlikely that many people will intensively withdraw treatment during this extremely short period. Therefore, the majority of those who responded with “I am withdrawal this treatment” can be assumed to have withdrawn from treatment prior to BP measurement. Additionally, the higher BP found in participants who withdrew from HTTx, as shown in Supplementary Table 1, can be inferred as an effect of withdrawal from treatment. Third, the address information used in this study was not collected immediately after the earthquake, but from the time of the baseline survey (2–5 years after the earthquake). If individuals moved across inland and coastal areas between the earthquake and the survey, the association between place of residence (inland or coastal area) and risk of withdrawal from HTTx may have been underestimated.

## Conclusions

This study showed that the risk of HTTx withdrawal was higher in the coastal areas (i.e., areas that were more severely affected by the tsunami) compared to the inland areas of Miyagi Prefecture. The study also showed that the risk of HTTx withdrawal was higher for participants whose homes were relatively less damaged among the coastal areas, while the risk of HTTx withdrawal for participants whose homes were more than half destroyed was similar to the inland areas. In addition, more participants who withdrew from HTTx had hypertension than those who were under HTTx. Those whose homes were more than half-destroyed may have been able to continue their treatment with government support such as through medical expenses exemption and being given a supply of ethical pharmaceuticals. Post-disaster administrative support for disaster victims, such as financial support, is considered vital for the prevention of the onset of CVD.

### Supplementary information

Supplementary information

Checklist
