# Supplementary material for: The risk of withdrawal from hypertension treatment in coastal areas after the Great East Japan Earthquake: the TMM CommCohort Study
Source: Hypertens Res. 2023 Oct 13;46(12):2718–28. doi: 10.1038/s41440-023-01454-0 (PMC10695828; doi:10.1038/s41440-023-01454-0)
Supplement: Supplementary file 1 — Supplementary information [file 41440_2023_1454_MOESM1_ESM.docx]

**Supplementary Figure 1.** Definition of inland and coastal areas


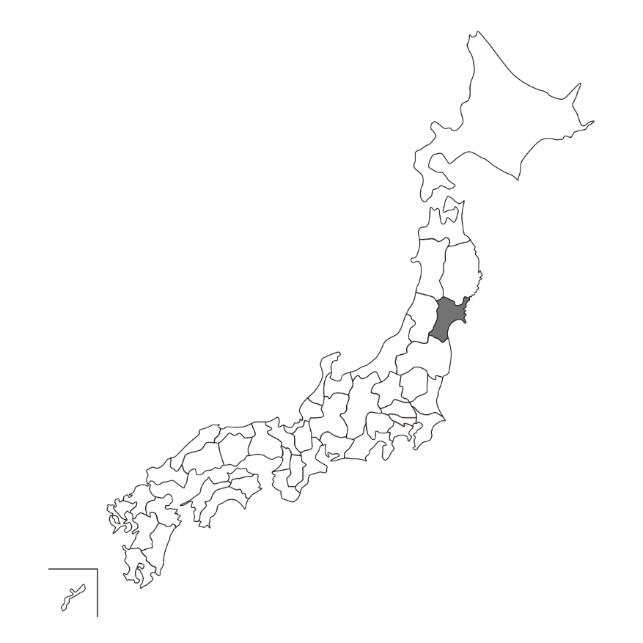

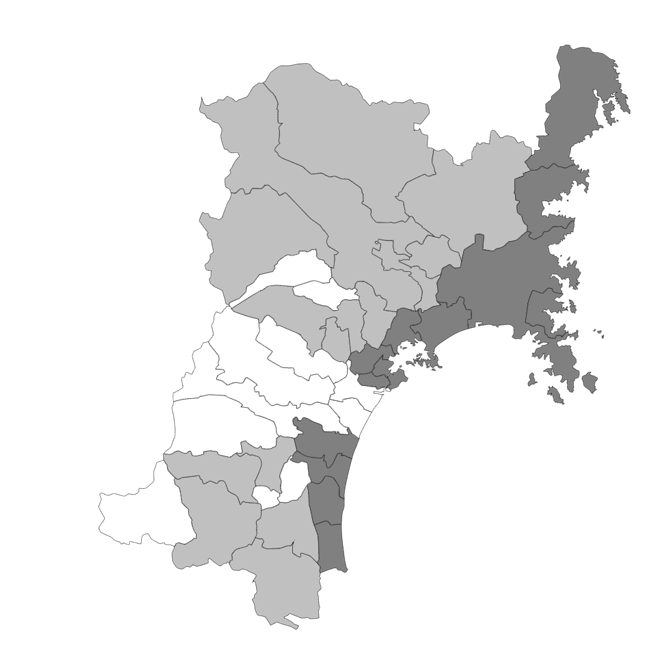


Miyagi Prefecture

Coastal areas

Inland areas

The 14 municipalities, including the sea-facing area, were defined as coastal areas (shown in dark gray). Another 14 municipalities were defined as inland areas (shown in light gray).

The names of the municipalities, divided into coastal and inland areas, are enumerated below. The order in which the municipalities are listed follows the order of the Ministry of Internal Affairs and Communications’ municipal codes (updated May 1, 2019).

Coastal areas: Ishinomaki City, Shiogama City, Kesennuma City, Natori City, Tagajo City, Iwanuma City, Higashimatsushima City, Watari Town, Yamamoto Town, Matsushima Town, Shichigahama Town, Rifu Town, Onagawa Town, Minamisanriku Town

Inland areas: Shiroishi City, Kakuda City, Tome City, Kurihara City, Osaki City, Tomiya City, Zao Town, Murata Town, Marumori Town, Taiwa Town, Osato Town, Kami Town, Wakuya Town, Misato Town

**Supplementary Table 1.** Comparison of SBP and DBP between participants under HTTx (n=8,878) and those withdrawing from HTTx (n=340)

|  | Under HTTx  (n=8,878) | Withdrawing from HTTx  (n=340) | P |
| --- | --- | --- | --- |
| Adjusted SBP  (mmHg） | 132.1  （131.8-132.4） | 141.0  (139.3-142.6) | <0.001 |
| Adjusted DBP  (mmHg） | 78.5  （78.3-78.7） | 84.5  (83.4-85.5) | <0.001 |

DBP, diastolic blood pressure; HTTx, treatment for hypertension; SBP, systolic blood pressure.

The values in the table are presented as means (95% confidence intervals).

Analysis of covariance (ANCOVA) was used to examine differences in the blood pressure levels between the groups (Under HTTx or Withdrawing from HTTx)

Adjusted for age, sex, body mass index, smoking status, drinking status, and estimated daily salt intake.
